# Lung epithelial and alveolar macrophage-like cell interactions significantly modify innate responses to bacterial endotoxin with the involvement of direct cellular contacts, TNF-α, ICAM1 and MCP-1

**DOI:** 10.3389/fimmu.2025.1715943

**Published:** 2026-01-09

**Authors:** Connor Wood, Shagun Khera, Minjeong Woo, Vikram Sharma, Justyna Lopatecka, Frederic Coulon, Zaheer Nasir, Vincent Delorme, Simon K. Jackson, György Fejer

**Affiliations:** 1 Institut Pasteur Korea, Tuberculosis Research Laboratory, Seongnam, Gyeonggi, Republic of Korea; 2 School of Biomedical Sciences, Faculty of Health, Plymouth University, Plymouth, United Kingdom; 3 Cranfield University, Faculty of Engineering and Applied Sciences, Cranfield, United Kingdom

**Keywords:** co-culture, epithelial cells, intercellular adhesion molecule 1 (ICAM-1), LPS, lung alveolar macrophages, MPI cells

## Abstract

**Introduction:**

Lung alveolar macrophages (AMs) and epithelial cells form the first line of defense against inhaled pathogens. Their interactions strongly influence innate immune responses in the lung, yet the mechanisms underlying this cross-talk remain incompletely understood.

**Methods:**

In this study, we established a co-culture system using a primary model of AMs (MPI alveolar macrophage-like cells) and MLE-12 alveolar epithelial cells to investigate innate responses and cellular interactions during bacterial lipopolysaccharide (LPS)-induced TLR4 activation.

**Results:**

Cytokine and chemokine profiling revealed that co-cultures exhibited significantly enhanced proinflammatory responses to both LPS and TLR2 ligands—including IL-6, TNF-a, and MCP-1 secretion—compared with mono-cultures. Strikingly, we identified MLE-12 epithelial cells as a source of lipopolysaccharide-binding protein (LBP), which is essential for LPS recognition in AMs and MPI alveolar macrophage-like cells. LBP secretion by epithelial cells explained cytokine responses to LPS under serum-free conditions; however, additional mechanisms—apparent in the presence of serum/LBP—also contributed to the amplified co-culture responses. These mechanisms included direct cell–cell contacts, as conditioned media from unstimulated cells failed to reproduce similar effects in mono-cultures. Moreover, co-cultures of naïve MPI cells and inflamed epithelial cells (MLE-12 cells pretreated with media from activated MPI macrophages) were found to release a nonnegligible amount of chemokines, even in the absence of LPS. This demonstrated an inflammatory amplification loop mediated by both contact dependent and soluble factors. Phospho-flow cytometry further revealed coculture- specific signaling, with enhanced MAPK pathway activation in macrophages and NF-kB activation in epithelial cells. Finally, LPS-activated MPI alveolar macrophage-like cells induced TNF-a–dependent ICAM-1 expression and apoptosis in MLE-12 cells. Increased ICAM-1 expression, in turn, promoted MCP-1 production in epithelial cells in an ICAM-1–dependent and cell contact mediated manner.

**Discussion:**

Together, these findings identify cellular contacts and a TNF-a–ICAM-1–MCP-1 axis—supported by epithelial-derived LBP—as key drivers of innate immune synergy between lung alveolar macrophages and epithelial cells. Our results establish the MPI–MLE-12 co-culture as a tractable model for dissecting pulmonary innate immune mechanisms.

## Introduction

Alveolar macrophages (AMs), the tissue-resident macrophages of the lung, represent the first line of defense against invading respiratory pathogens. They constitute a unique subset of macrophages characterized by distinct immune signaling pathways and cytokine secretion profiles (1), likely reflecting their specialized developmental origin and growth factor requirements. Unlike most other macrophage populations that depend on macrophage colony-stimulating factor (M-CSF), AMs differentiate and are maintained by granulocyte-macrophage colony-stimulating factor (GM-CSF), which is constitutively produced in the lung (2–4).

In contrast to inflammatory macrophages and certain tissue-resident macrophages that have limited lifespans and are replenished by bone marrow-derived monocytes, AMs originate from fetal liver monocytes and are self-renewing within the lung environment (5, 6). While bone marrow-derived macrophages can be generated *in vitro* in substantial numbers (7), AMs remain scarce and require invasive procedures for isolation (8). This limits the study of their unique innate immune responses.

Alveolar epithelial cells also influence the differentiation and function of both resident and recruited immune cells (9). Type I alveolar epithelial cells primarily contribute to gas exchange and lung structure (10), whereas Type II alveolar cells produce secreted factors—such as surfactant proteins and phospholipids—that shape the unique immunological milieu of the alveolar space. These factors are believed to play a key role in the differentiation and activation of AMs (11, 12). Moreover, cross-talk between AMs and alveolar epithelial cells is essential for maintaining immune homeostasis in the lung (13).

Previous research on the interaction between macrophages and alveolar epithelial cells has highlighted the transmembrane glycoprotein intercellular adhesion molecule-1 (ICAM-1) as a crucial mediator of immune responses, especially to lipopolysaccharide (LPS) stimulation (14, 15). ICAM-1 is involved in endothelial transmigration, intracellular signaling, cell-cell interactions, and tissue integrity (16, 17). It acts as a specific ligand for leukocyte function-associated antigen-1 (LFA-1) and is upregulated under inflammatory conditions (17).

Recently, a novel *in vitro* model of AMs known as MPI alveolar macrophage-like cells has been developed. These are primary, non-transformed, continuously proliferating GM-CSF-dependent macrophages that accurately recapitulate the distinctive responses of AMs and are available in unlimited quantities. MPI cells have already been employed to investigate responses to various bacterial and viral respiratory pathogens (18–21).

In this study, we established a co-culture model of MPI alveolar macrophage-like cells and MLE-12 alveolar epithelial cells—a widely used murine model of Type II alveolar cells (22)—to investigate their cross-talk during bacterial LPS stimulation. We demonstrate that co-culture with MLE-12 cells induces a distinct immune activation profile in MPI alveolar macrophage-like cells, characterized by enhanced cytokine production and epithelial cell death. Our findings further suggest that direct cell contact and ICAM-1 expression significantly contribute to these effects.

## Materials & methods

### Cell culture

MPI cells were grown at 37°C, 5% CO_2_ in RPMI 1640 medium (Welgene) supplemented with 10% heat-inactivated fetal bovine serum (FBS, Gibco – Endotoxin <0.05 EU/mL), referred to as MPI medium.

MPI cells were maintained in RPMI-FBS and 10 ng/mL GM-CSF and passaged twice a week at a concentration of 2 × 10^5^ cells/mL. Cells from passages 8 (P8) to 70 (P70) were used.

Constitutively fluorescent, hCD68^GFP^ MPI cells were established from hCD68^GFP^ reporter mice, acquired from The Jackson Laboratory (C57BL/6-Tg(CD68-EGFP)1Drg/J; strain number 026827). Homozygous males were mated individually with C57BL/6 females and MPI cells were established from the liver of fetuses, as described in (18). These GFP-expressing MPI cells were used only in experiments where separate analysis of MPI and MLE-12 cell populations within the co-culture was required. For all other experiments involving the co-culture system, unlabeled MPI cells from the same lineage were used.

MLE-12 cells (mouse lung type II epithelial cell line) were obtained from the American Type Culture Collection (CRL-2110; ATCC, Manassas, VA, USA). MLE-12 cells were grown as monolayer cultures at 37°C, 5% CO_2_ in Dulbecco’s medium:Ham’s F12, 50/50 (Gibco) supplemented with 2% heat-inactivated fetal bovine serum (FBS, Gibco), Insulin-Transferrin-Selenium (Gibco), 10 nM Hydrocortisone (Sigma-Aldrich), and 10 nM β-estradiol (Sigma-Aldrich), referred to as MLE-12 medium. Cells were passaged twice a week at a concentration of 5 × 10^4^/mL and used from passage 8 (P8) to 50 (P50).

Murine bone-marrow was obtained by flushing femurs of 6- to 8-week-old Balb/c mice (Orient Bio) with RPMI-FBS. The recovered cells were differentiated into bone marrow-derived macrophages (BMMs) by exposure to 40 ng/mL recombinant murine macrophage colony-stimulating factor (M-CSF, Miltenyi Biotec) for 7 days. These BMMs were used only for experiments requiring a primary macrophage comparator and were not employed in the MPI/MLE-12 co-culture assays.

### MPI/MLE-12 co-culture

For co-culture, MLE-12 cells were seeded at a density of 2 × 10^5^/mL one day prior to macrophage addition to form a confluent monolayer. MPI alveolar macrophage-like cells, resuspended in MLE-12 medium, were then added to this monolayer at the indicated experimental densities and the co-culture incubated for 24h (unless otherwise stated) at 37°C, 5% CO_2_.

For indirect co-cultures, MLE-12 cells were seed at a density of 1x10^5^/mL and allowed to grow to confluency over 3 days incubated at 37°C, 5% CO_2_. The supernatant was collected and filtered through a 0.2 μm syringe filter before being applied to pre-seeded MPI cells.

### Lipopolysaccharide binding protein quantification

Supernatants from mono- and co-cultures seeded at different densities were analyzed for LBP production using ELISA (biometec, GmbH) following manufacturer’s instructions. Results were expressed as ng/ml.

### Cell stimulation

Cells were stimulated with 100 ng/mL of Ultrapure LPS from *E. coli* 0111:B4 (Invivogen) for 24h, unless otherwise stated. For generation of LPS activated MPI condition media, MPI cells seeded at a density of 5 × 10^5^/mL were stimulated with 100 ng/ml LPS for 24h. The supernatant was collected and filtered through a 0.2 μm syringe filter prior to treatment with 250 μg/mL Polymyxin B for 15 min to inactivate the LPS. MLE-12 cells were then incubated with this medium and referred to as inflammatory MLE-12 (MLE-12 *inflam*) or non-LPS treated control medium for 24h. For generation of unstimulated MPI condition media, preparation was performed identically as above, with the omission of LPS.

For generation of activated LPS MLE-12 condition media, preparation was performed as above, with a cell density of 2x10^5^/mL. This media was also pre-treated with 250 μg/mL Polymyxin B for 15 min to inactivate the LPS, prior to incubation with MPI cells.

For generation of unstimulated MLE-12 condition media, preparation was performed identically as above, with the omission of LPS.

In experiments involving pre-incubation with these activated condition media or non-activated condition media prior to a secondary LPS stimulation, the condition media was removed, and cells washed three times in PBS to remove residual Polymyxin B or cell derived factors before subsequent LPS stimulation.

Response to FSL-1 (Invivogen) and Cockroach Extract (Greer Laboratories) was determined by exposing the cells to varying concentrations of these stimulants for 24h.

### ICAM-1 and TNF-α neutralization

For ICAM-1 neutralization, MLE-12 monolayers were incubated with 5 µg/mL of ICAM-1 blocking antibody (Mouse ICAM-1/CD54, R&D Systems) for 1h prior to addition of MPI cells, as previously reported (23).

For TNF-α neutralization MPI condition media was prepared as described above with the addition of TNF-α blocking antibody (InVivoMAb anti-mouse TNF-α (Bio X Cell) at 25 µg/mL for 1h prior to the addition to the MLE-12 monolayer, as previously reported (24).

### Fluorescence activated cell sorting

For antibody-staining, cells were first fixed in 4% formalin and Fc receptor mediated binding was reduced by incubation with Anti-mouse CD16/CD32 (Mouse BD Fc Block™, BD Bioscience).

For ICAM-1 surface expression, cells were incubated with monoclonal rabbit Anti-mouse ICAM-1 (Abcam, EPR22161-284) for 2h, at a dilution of 1:100, followed by a secondary incubation with anti-rabbit IgG Alexa Fluor™ 647 (Invitrogen) for 45 min, at a dilution of 1:2000.

For phospho – ERK, JNK, p38, and NF-κB expression, cells were permeabilized with 0.1% Triton X-100 for 10 min prior to blocking with Anti-mouse CD16/CD32 (Mouse BD Fc Block™, BD Bioscience), then incubated with monoclonal rabbit Phospho-p44/42 MAPK (Erk1/2) (Thr202/Tyr204) (D13.14.4E), mouse Phospho-SAPK/JNK (Thr183/Tyr185) (G9), mouse Phospho-p38 MAPK (Thr180/Tyr182) (28B10), or rabbit Phospho-NF-κB p65 (Ser536) (93H1) (Cell Signaling technologies) for 2h, at a dilution of 1:500. A secondary incubation was then performed with either anti-rabbit or mouse IgG Alexa Fluor™ 647 (Invitrogen) or 45 min, at a dilution of 1:2,000.

To distinguish MPI and MLE-12 cells in co-cultures an initial gating strategy was established using GFP expressing MPI and non-expressing MLE-12 cells, without antibody staining, to separate the hCD68^GFP^ MPI cells from MLE-12 cell population (**Supplementary Figure S2A**).

For apoptotic cell determination, live cells were stained using CellEvent™ Caspase-3/7 Green Flow Cytometry Assay Kit (Invitrogen) following the manufacturer instructions.

Flow cytometry data was captured via CytoFLEx LS (Beckman Coulter) with a minimum of 10,000 events captured after isolation of single cell populations using SSC and FSC gating to remove debris and doublets (**Supplementary Figure S2B**). For Alexa Fluor™ 647 detection R660-APC channel was utilized (ex: 638, em: 660/10), for GFP and Caspase-3/7 Green Detection Reagent detection B525-FITC channel was utilized (ex: 488, em: 525/40), for SYTOX™ AADvanced™ Dead Cell Stain detection B690-PC5.5 channel was used (ex: 488, em: 690/50).

### Cytokine ELISA

Cell supernatants were collected and secreted cytokines were quantified using DuoSet ELISA kits (R&D systems), following the manufacturer instructions.

### Luminex assay

MLE-12 and MPI cells were co-cultured at a ratio of 10:1 in 96-well flat bottom tissue culture plates as described previously. Cells were stimulated with 100 ng/mL of Ultrapure LPS from *E. coli* 0111:B4 (Invivogen) for 24h. Supernatants were collected and Luminex assay was performed using MILLIPLEX^®^ MAP kit for the detection of multiple analytes (Merck Millipore). These included IL-1α, IL12p70, IL-6, MIP-1α, MIP-1β, MCP-1, MIP-2, RANTES, TNFα. Data was analyzed using MILLIPLEX^®^ Analyst 5.1 Software and expressed as pg/mL.

### Intracellular cytokine detection

After 4h of LPS stimulation 1 µg/mL of Brefeldin A was added for an additional 3h to block cytokine release. Cells were then fixed in 4% paraformaldehyde and permeabilized with 0.1% Triton X-100 for 10 min prior to blocking with Anti-mouse CD16/CD32 (Mouse BD Fc Block™, BD Bioscience), then incubated with monoclonal rabbit MCP1 (EPR27464-89) for 2h, at a dilution of 1:1,000, followed by a secondary incubation with anti-rabbit IgG Alexa Fluor™ 647 (Invitrogen) for 45 min, at a dilution of 1:2,000. Cells were counterstained with Hoechst 33342. Representative images were acquired by fluorescence microscopy using a 63X water objective (Opera Phenix, Revvity). For quantification a minimum of 2,000 cells across twenty-five fields were analyzed using the Signal Image Analysis system (Revvity), to quantify cells (Hoechst 33342), MPI (GFP), and MCP-1 (Alexa Fluor™ 647).

### Statistical analysis

Data were processed and visualized using GraphPad Prism (version 10.6.1). Pairwise comparisons were performed using unpaired Student’s t-test. For experiments involving multiple comparisons to a single control group, one-way ANOVA with Dunnett’s *post hoc* test was used. For analyses involving multiple comparisons across independent time points or experimental conditions, two-way ANOVA with Sidak’s *post hoc* test was applied. All flow cytometry data were processed and visualized using FlowJo (version 10).

## Results

### LPS-induced cytokine responses are increased in MPI/MLE-12 co-culture in the absence of FBS

**Figure 1:**
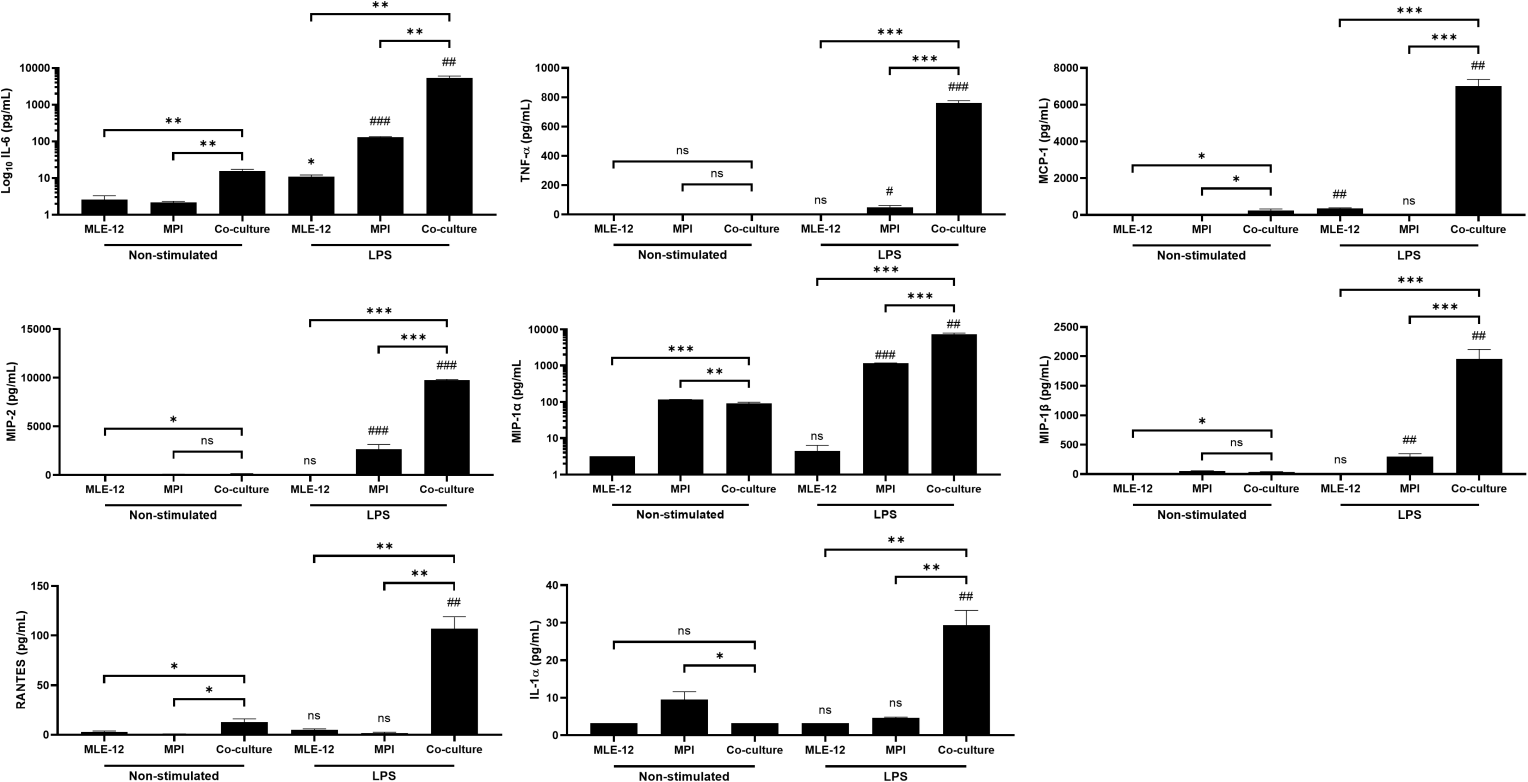
Co-culture of MLE-12 and MPI macrophages increases the production of various LPS stimulated cytokines. Production of IL-6, TNF-α, MCP-1, MIP-2, MIP-1α, MIP-1β, RANTES, and IL-1α in response to 100 ng/mL LPS in MLE-12 and MPI mono-cultures or co-cultures containing both cell types. Cells were stimulated with LPS and cytokines were quantified at 24h post treatment via LUMINEX. Data are representative of three independent experiments and expressed as mean ± SD for three biological replicates. Statistical significance between non-stimulated and LPS stimulated cells was determined by Prism software using the unpaired Student’s t-test. ^#^p-value < 0.05; ^##^p-value < 0.01; ^###^p-value < 0.001. *ns* was used to indicate when the differences were found to be non-significant. Additional pairwise comparison between monocultures and co-cultures was performed and the statistical significance was determined by Prism software using the One-Way ANOVA followed by Dunnett’s *post hoc* test. *p-value < 0.05; **p-value < 0.01; ***p-value < 0.001. ns was used to indicate when the differences were found to be non-significant.

We used the direct co-culture system described above (MPI and MLE-12 cells in the same well) to study LPS-induced production of multiple pro-inflammatory cytokines and chemokines under FBS-free conditions, and compared these responses with those from mono-cultures. As shown in **Figure 1**, although in some cases there were slight increases in the pro-inflammatory cytokine secretions in non-stimulated co-cultures, a strong clear increase in secretion was seen for the co-culture after the LPS challenge for all the measured secreted factors. Importantly, we found that MPI cells grown in mono-culture secreted only low levels of these inflammatory cytokines in response to LPS treatment.

At the core of this work was the establishment of a robust protocol allowing the probing of cellular cross-talks between alveolar epithelial and alveolar macrophage-like cells. A careful examination of multiple parameters was performed to evaluate variabilities arising from the medium, seeding conditions and co-culture time. In particular, while MPI cells were normally maintained in MPI medium, they had to be transferred in MLE medium during the co-culture experiments. The effect of such a media change on MPI cells was assessed beforehand, leading to no observable change in cytokine secretions in presence or absence of lipopolysaccharide (LPS) (**Supplementary Figure S1**).

### LPS stimulated IL-6 production in co-culture increase is dependent on LPS dose and macrophage numbers

**Figure 2:**
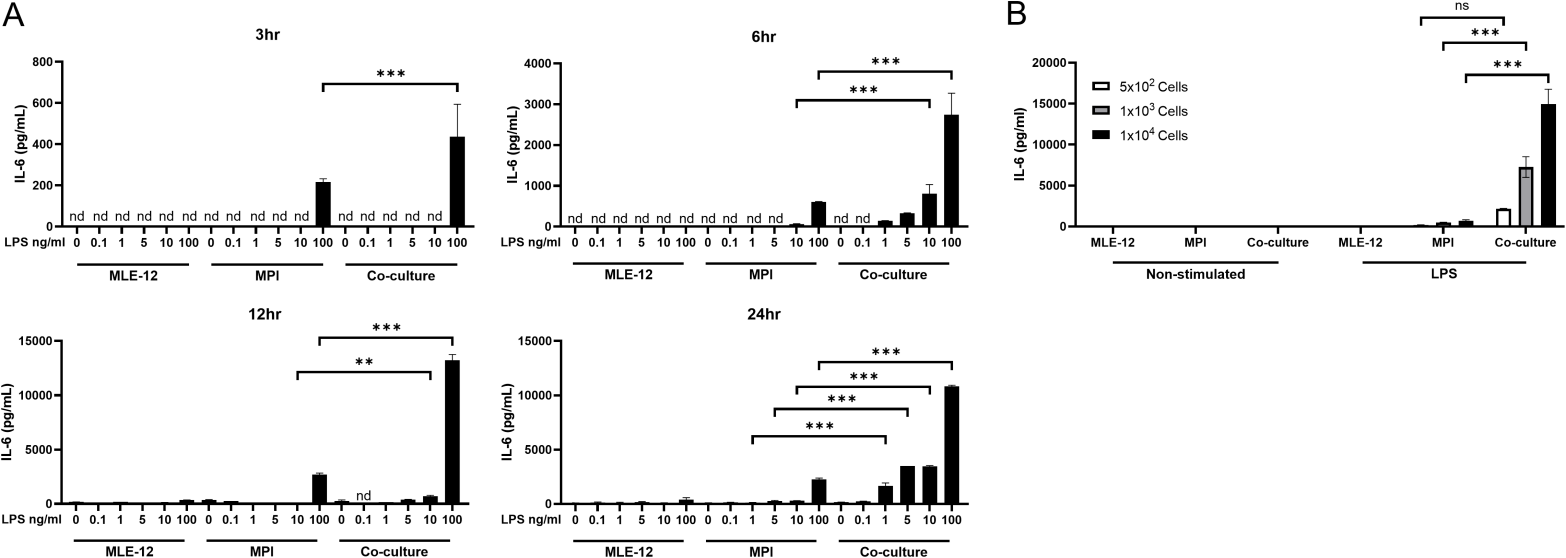
LPS-stimulated IL-6 production increase in co-culture is dose-dependent and related to MPI cell number. Production of IL-6, in response to increasing doses of LPS in MLE-12 and MPI isolated cultures or co-cultures containing both cell types **(A)**. Cells were stimulated with LPS and IL-6 secretion was quantified at 24h post treatment via ELISA. Production of IL-6, in response to LPS in increasing numbers of MPI cells in isolated cultures or co-cultures containing both cell types **(B)**. Cells were stimulated with LPS and IL-6 was quantified at 24h post treatment via ELISA. Statistical significance between MPI mono-cultures and co-cultures were determined by Prism software using the 2-way ANOVA followed by Šídák’s *post hoc* test. **p-value < 0.01; ***p-value < 0.001. Nothing was indicated when the differences were found to be non-significant. nd was used to indicate no detectable value.

LPS stimulated IL-6 production in a dose- and time-dependent manner in the co-culture (**Figure 2A**), with higher levels of IL-6 produced at 24hrs post stimulation vs 3hrs and 100 ng/mL vs 0.1 ng/mL LPS stimulation. The numbers of MPI alveolar macrophage-like cells in the co-culture model also contribute to the IL-6 levels following LPS stimulation (**Figure 2B**).

### MLE-12 cells produce LBP

**Figure 3:**
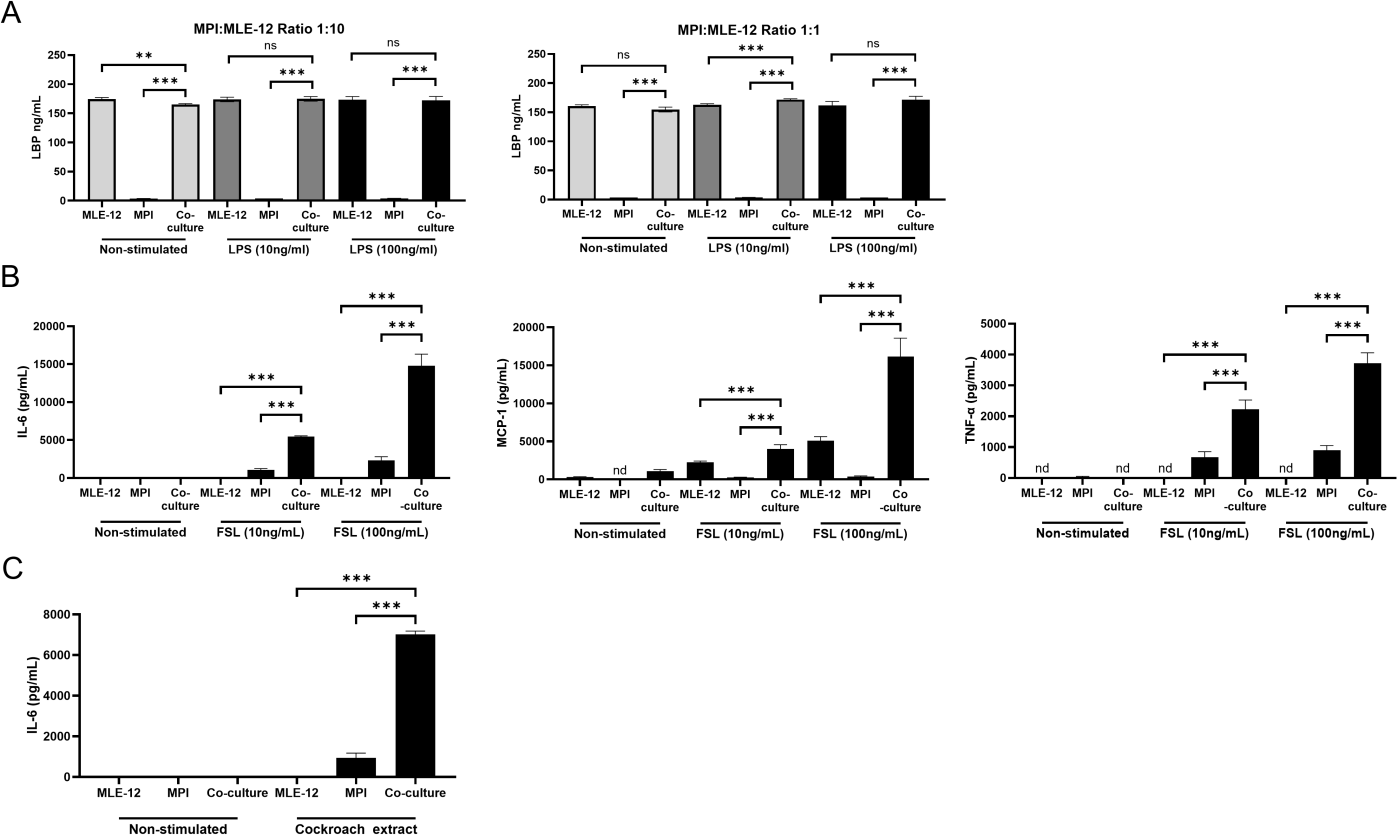
MLE-12 cells produce LBP for enhanced co-culture LPS response and co-culture of lung epithelial cells and MPI macrophages increases the production of LBP independent TLR2 and allergen stimulated cytokines. LBP production in MLE-12 and MPI cultures, or co-cultures containing both cell types, when left unstimulated or after exposure to LPS **(A)**. Cells were stimulated with LPS and LBP was quantified at 24h post treatment via ELISA. Production of IL-6, MCP-1, and TNF-α in response to FSL-1 (Pam2CGDPKHPKSF) **(B)** or IL-6 production in response to Cockroach extract allergen **(C)**. Cells were stimulated and cytokines were quantified at 24h post treatment via ELISA. Statistical significance between mono-cultures and co-cultures were determined by Prism software using the One-Way ANOVA followed by Dunnett’s *post hoc* test. **p-value < 0.01; ***p-value < 0.001. ns was used to indicate when the differences were found to be non-significant.

MPI cells are strictly dependent on LBP for LPS stimulated cytokine production (18), however, in the absence of serum/LBP, LPS stimulated high levels of cytokine production the co-culture (**Figure 1**). An investigation of the LBP secretions revealed that the MLE-12 cells were solely responsible for the production of LBP and that they were able to secrete it in the co-culture condition as well (**Figure 3A**).

### Non-LBP dependent mechanisms also contribute to enhanced cytokine responses in MPI/MLE-12 co-cultures

The detection of MLE-12 secreted LBP prompted us to examine the effects of the co-cultivation on the immune responses following stimulation by other LBP-independent ligands, such as the TLR-2/6 agonist FSL-1 lipopeptide and the allergen cockroach extract, described as a TLR2 stimulus as well (25).

As expected, these stimuli induced cytokine production even in the absence of FBS in MPI cells alone. Interestingly, the pro-inflammatory cytokine levels induced by these stimulants were again significantly enhanced in the co-cultures compared to the MPI cells alone (**Figures 3B, C**). These results suggest that, although the secretion of LBP by MLE-12 cells could be an important factor for the enhanced response to LPS, additional mechanisms were at work for the enhancement of the immune responses independently of LBP in the alveolar macrophage-like cell/epithelial cell co-culture.

### LPS responses in co-cultures are enhanced independent of LBP and direct contact of MLE-12 and MPI cells is required for this response

**Figure 4:**
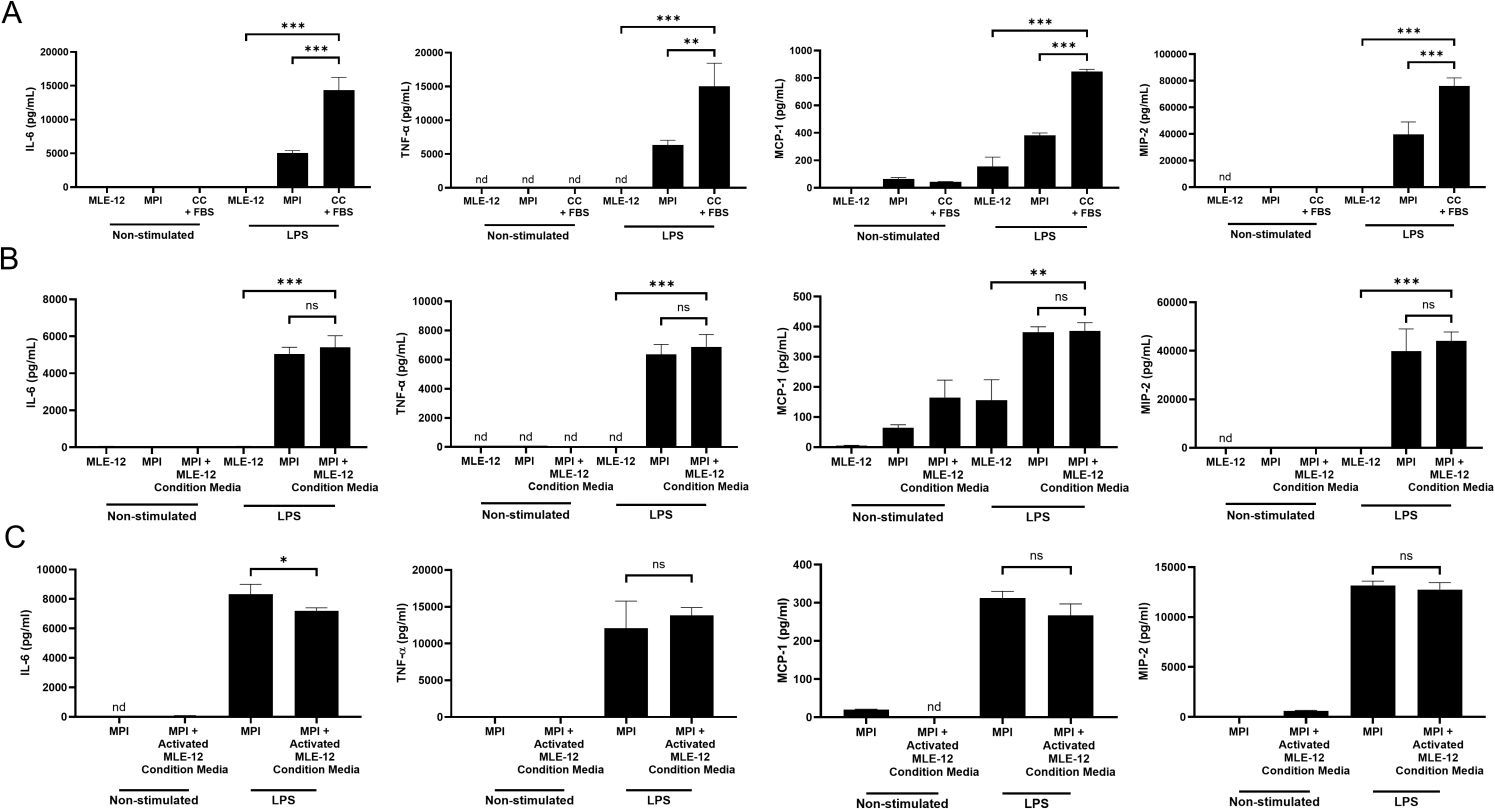
Direct cell to cell contacts rather than transfer of soluble factors is required for the synergistic cytokine production effects in co-culture. Production of IL-6, TNF-α, MCP-1, and MIP-2 in response to 100 ng/mL LPS in MLE-12 and MPI cell cultures or direct co-cultures (CC) containing both cell types in the presence of FBS (10%) **(A)**, with condition media from MLE-12 cells **(B)**, and condition media from LPS activated MLE-12 cells **(C)**. Cells were stimulated with LPS and cytokines were quantified at 24h post treatment via ELISA. Data are representative of three independent experiments and expressed as mean ± SD for three biological replicates. Statistical significance between isolated cultures and co-cultures **(A, B)** were determined by Prism using the One-Way ANOVA followed by Dunnett’s *post hoc* test. *p-value < 0.05; **p-value < 0.01; ***p-value < 0.001. *nd*, not detectable. *ns* was used to indicate when the differences were found to be non-significant. nd was used to indicate no detectable value. Statistical significance between MPI and MPI with conditioned media **(C)** were determined by Prism software using the unpaired Student’s t-test. *p-value < 0.05. *nd*, not detectable. *ns* was used to indicate when the differences were found to be non-significant. nd was used to indicate no detectable value.

To investigate the possible LBP-independent LPS stimulation enhancement mechanisms further, we studied the inflammatory responses to LPS in the presence of FBS (thus LBP) containing media using a 24h pre-stimulation co-culture incubation period. Under this condition, the co-cultured cells were able to secrete significantly more inflammatory cytokines compared to the cells grown in mono-culture, although the increase was not as strong as what could be observed in medium lacking FBS (**Figures 1**, **2**). A shorter co-culture of 3hrs was not sufficient to enhance LPS responses (**Supplementary Figure S2**). To determine whether the enhancement observed in the co-culture (**Figure 4A**) was driven by effectors secreted by MLE-12 cells, we cultured MPI cells with conditioned medium from non-stimulated MLE-12 cells instead of performing a true co-culture (**Figure 4B**). Under these conditions, we detected no enhancement of LPS-stimulated cytokine production. Furthermore, adding conditioned medium from LPS-stimulated MLE-12 cells—after depleting LPS with polymyxin B—also failed to significantly increase cytokine expression in MPI cells (**Figure 4C**).

### MLE-12 cells express increased levels of ICAM-1 in co-culture with MPI cells upon LPS stimulation

**Figure 5:**
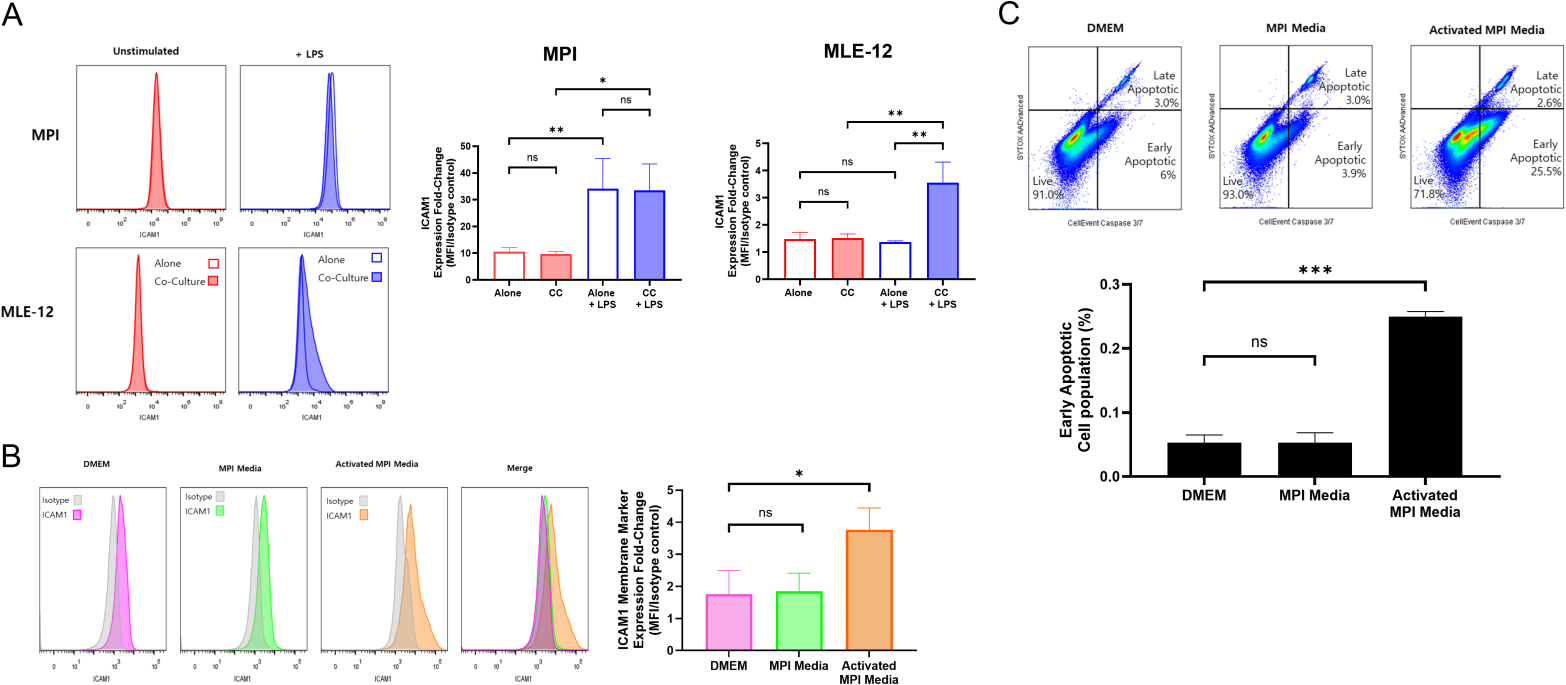
MLE-12 cells express increased levels of ICAM-1 when in co-culture with MPI cells or in medium conditioned with MPI cell secretions, which also induce apoptosis. **(A)** ICAM-1 expression levels in hCD^68^GFP MPI and MLE-12 cell cultures and co-cultures when stimulated with LPS. Cells were stimulated with LPS for 24h and stained for ICAM-1 expression, with cell populations being differentiated by constitutive GFP expression of MPI cells. **(B)** ICAM-1 expression levels in MLE-12 exposed to media from MPI or LPS-stimulated MPI cells (Activated MPI media) or to regular MLE culture DMEM media. Polymyxin B was added to all conditions, including DMEM and MPI media, to neutralize residual LPS and ensure proper normalization across treatments. All conditions contained polymyxin B to ensure correct normalization across treatments. **(C)** induction of apoptosis in MLE-12 cells when exposed to media from MPI or LPS stimulated MPI cells. MLE-12 cells were incubated in media conditioned by MPI cells, naive or stimulated with 100 ng/mL LPS, for 24h and stained for ICAM-1 expression. Cell populations were differentiated by GFP expression of hCD^68^GFP MPI cells. Histograms are representative of three independent experiments and bar charts are expressed as mean ± SD for three biological replicates. Pairwise comparisons were determined by Prism software using the One-Way ANOVA followed by Dunnett’s *post hoc* test. *p-value < 0.05; **p-value < 0.01; ***p-value < 0.001. ns was used to indicate when the differences were found to be non-significant.

Since ICAM-1 can mediate interactions between epithelial cells and leukocytes during inflammation (26), we decided to investigate more in detail the expression of this glycoprotein at the surface of the cells co-cultivated in our system. In order to distinguish the MPI cells from the MLE-12 cells, we used a constitutively fluorescent, hCD68^GFP^ MPI cell line using FACS (**Supplementary Figure S4**). These fluorescent MPI cells were co-cultivated with MLE-12 cells and the entire population was labelled with ICAM-1 antibodies for the analysis of the surface expression of this protein using FACS. In contrast to MPI cells that showed high basal levels of ICAM-1, MLE-12 cells were mostly negative for ICAM-1 expression in the absence of LPS stimulation either alone or in co-culture with MPI cells (**Figure 5A**) (**Supplementary Figure S5**). While LPS stimulation increased ICAM-1 expression on the surface of MPI cells, no changes were seen for MLE-12 cells cultivated alone. However, when the two different cell types were co-cultured and stimulated with LPS, a clear increase in ICAM-1 expression was seen for MLE-12 cells, as compared to the unstimulated control co-culture (**Figure 5A**).

### ICAM-1 expression in MLE-12 cells is induced by soluble factors secreted by MPI cells

As the increase in ICAM-1 expression by MLE-12 cells was only seen in co-culture, we investigated whether this was due to a factor secreted by the MPI cells. To this end, we used conditioned media from MPI cells to culture MLE-12 cells. When applying medium conditioned by naïve MPI cells to MLE-12 cells, no increase in ICAM-1 expression was observed (**Figure 5B**). However, ICAM-1 expression was significantly increased when applying a medium conditioned by MPI cells treated with LPS, which was then LPS depleted with polymyxin B (LPS-activated MPI media), (**Figure 5B**). We also noted that the LPS-activated MPI medium was inducing morphological changes in the MLE-12 cells (**Supplementary Figure S6**), more specifically an increase in cell shrinkage and membrane blebbing, suggestive for apoptosis. Apoptosis was therefore quantified by FACS analysis using a Caspase 3/7 labelling strategy, revealing a significant increase in the number of apoptotic cells when incubating the MLE-12 in LPS-activated MPI media, as compared to the control (**Figure 5C**). These results indicated that soluble factors secreted by LPS-stimulated MPI cells could be responsible for the ICAM-1 surface expression and apoptosis of MLE-12 cells.

### TNF-α secretion by MPI cells induces ICAM-1 and apoptosis in MLE-12 cells

**Figure 6:**
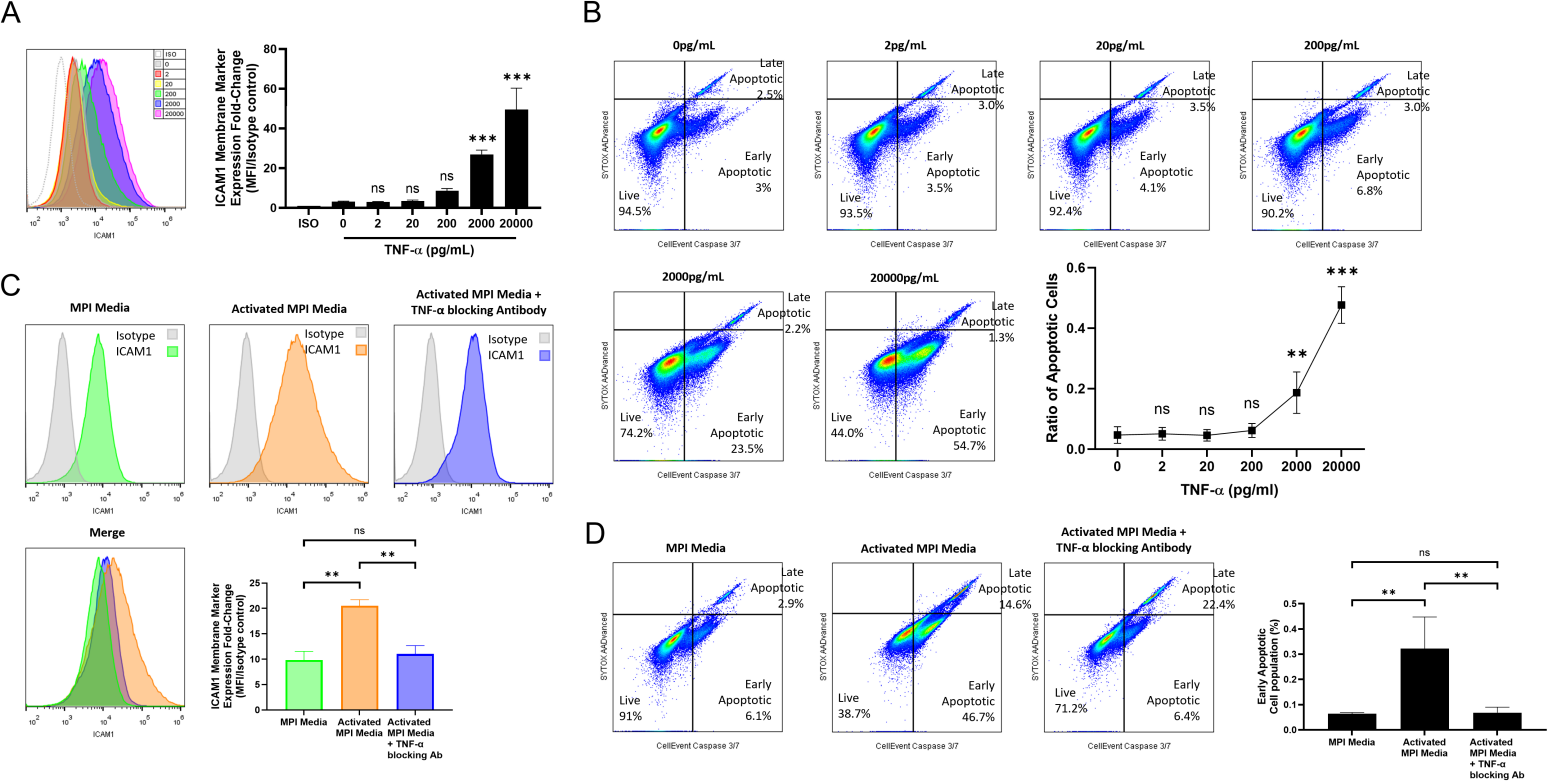
TNF-α secreted from MPI cells induces ICAM-1 expression and apoptosis in MLE-12 cells. Effect of recombinant TNF-α on ICAM-1 expression **(A)** and apoptosis **(B)** in MLE-12 cells. MLE-12 cells were incubated with increasing concentrations of recombinant TNF-α for 24h. Effect of TNF-α inhibition in MPI secretions on ICAM-1 expression **(C)** and apoptosis **(D)** in MLE-12 cells. Polymyxin B was added to all conditions, including MPI media, to neutralize residual LPS and ensure proper normalization across treatments. Histograms are representative of three independent experiments and bar charts are expressed as mean ± SD for three experimental replicates. Multiple pairwise comparisons were performed by Prism software using One-Way ANOVA followed by Dunnett’s *post hoc* test. **p-value < 0.01; ***p-value < 0.001. ns was indicated when the differences were found to be non-significant.

As TNF-α is a cytokine predominantly secreted by macrophages in response to LPS stimulation (27) and is well known for induction of apoptosis (28), we decided to explore the effects of recombinant TNF-α on the MLE-12 cells. Firstly, we found that exposure of MLE-12 cells to TNF-α was able to induce apoptosis in a dose-dependent manner, with concentrations of 2,000 and 20,000 pg/mL able to trigger apoptosis in 23.5% and 54.7% of the MLE-12 cells, respectively (**Figure 6B**). Secondly, we observed that TNF-α was able to increase the expression of ICAM-1 at the surface of MLE-12 cells (**Figure 6A**).

The specific role of TNF-α to induce ICAM-1 expression and elicit MLE-12 cell apoptosis was further confirmed by the use of TNF-α blocking antibodies added to the LPS-activated MPI media prior to incubation with the MLE-12 cells. Inhibition of TNF-α in this media prevented any increase in ICAM-1 expression and also inhibited the induction of apoptosis in the MLE-12 cells (**Figures 6C, D**).

Collectively, these data indicate that soluble TNF-α secreted by MPI cells in response to LPS treatment was most likely playing an important role in the response observed with MLE-12 cells.

### MPI cells show increased MAP kinase and MLE-12 cell enhanced NF-κB activation in co-culture upon LPS stimulation

**Figure 7:**
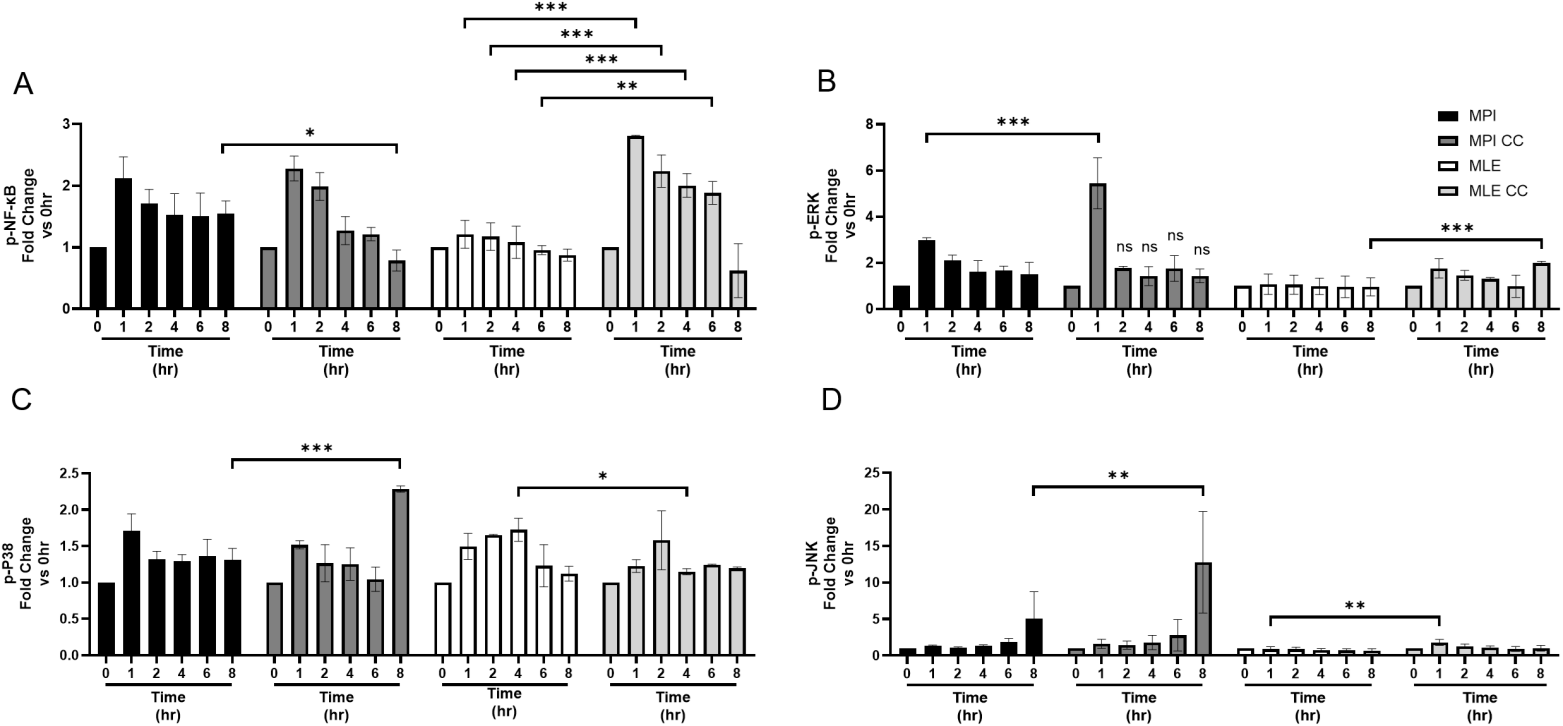
LPS stimulation of co-cultures induces stronger MAP kinase or NF-κB activation in MPI cells and MLE-12 epithelial cells, respectively. Phosphorylation of NF-κB **(A)**, ERK **(B)**, P-38 **(C)**, and JNK **(D)** were measured by flow cytometry after 100 ng/ml LPS stimulation for indicated time points. The hCD^68^GFP MPI cells were used to discriminate MPI from MLE-12 cells background when in co-culture, based on GFP expression. Data are a combination of three independent experiments and expressed as mean ± SD. Multiple pairwise comparisons for cells in monocultures or co-cultures were performed by Prism software using the 2-way ANOVA followed by Šídák’s *post hoc* test. *p-value < 0.05; **p-value < 0.01; ***p-value < 0.001. nothing was indicated when the differences were found to be non-significant.

The activation of NF-κB and MAP kinases plays key roles in LPS triggered activation of cytokine production. Therefore, we tested the activation of these factors to LPS in the co-culture system. We used the hCD^68^GFP MPI cells to discriminate between MPI and MLE-12 cells and assessed the changes induced by LPS to the different cellular effectors using FACS. MPI cells, when in co-culture, showed a late increase (8h) of P38 and JNK activation (**Figures 7C, D**), while an early increase (1h) of ERK activation was also observed, as compared to mono-cultures (**Figure 7B**) but changes of NF-κB activation was not observed between mono- and co-cultures in MPI cells. In contrast with MLE-12 cells, there was a significant increase in NF-κB activation from 1 to 4h following LPS stimulation when the cells were cultivated in the co-culture system (**Figure 7A**) but no changes in MAP kinase activation was found for MLE-12 cells in mono- versus co-culture.

### Activated MLE-12 cells are able to induce chemokine production when co-cultured with naïve MPI cells

**Figure 8:**
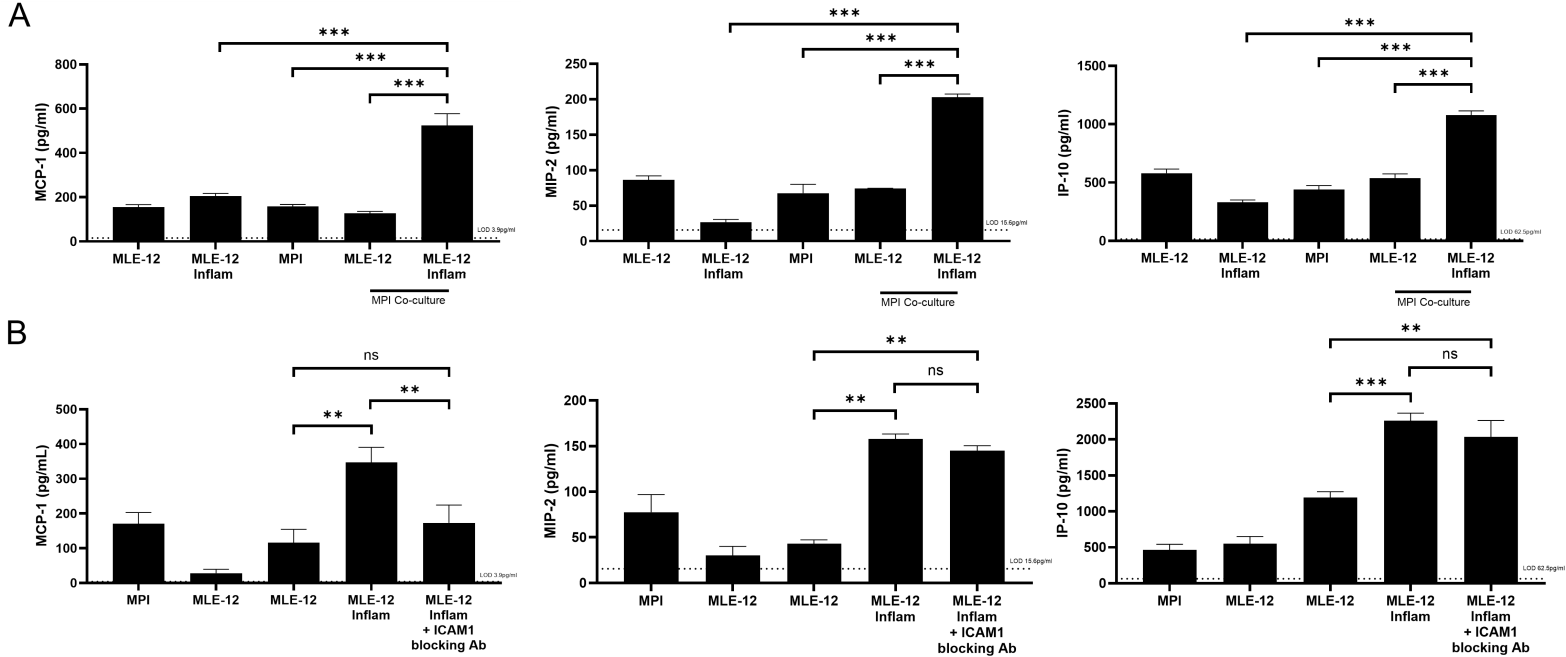
MLE-12 cells exposed to LPS-activated MPI cells supernatant can in turn induce a chemokine response in naïve MPI cells, even in the absence of other inflammatory stimulants, with MCP-1 being dependent on ICAM-1. Induction of MCP-1, MIP-2 and IP-10 in co-cultures of naïve MPI cells with MLE-12 cells exposed to LPS-activated MPI cells supernatants, or inflammatory MLE-12 cells (MLE-12 *Inflam*), in the absence of additional external stimuli **(A)**. MCP-1 production in co-cultures of naïve MPI cells and MLE-12 *Inflam*, or MLE-12 *Inflam* treated with ICAM-1 blocking antibodies **(B)**. Cells were cultured together and cytokines were quantified after 24h via ELISA. Data are representative of three independent experiments and expressed as mean ± SD for three biological replicates. Multiple pairwise comparisons were performed by Prism software using One-Way ANOVA followed by Dunnett’s *post hoc* test. **p-value < 0.01; ***p-value < 0.001. ns was indicated when the differences were found to be non-significant.

To study the mechanisms behind the changed cytokine responses of co-cultures to LPS, we exposed activated MLE-12 cells to naïve MPI cells to study the impact on cytokine secretion. To this end, MLE-12 cells were first exposed to the LPS-activated MPI medium (culture supernatant of LPS-treated MPI cells) to obtain inflammation activated MLE-12 cells (MLE-12 *Inflam*), followed by washing and addition of naïve MPI cells to this monolayer of MLE-12 *Inflam* cells. Significant increase in the secretion of MCP-1, MIP-2 and IP-10 (**Figure 8**) but not of IL-6 and TNF-α (**Supplementary Figure S7**) were observed after 24h of co-culture, as compared to co-cultures with naïve MLE-12 cells or MPI cells alone (**Figure 8A**). Importantly, no LPS addition was required to see this effect, indicating that MPI cells can produce chemokines solely with contact with activated MLE-12 cells. Since ICAM-1 was induced in MLE12 cells in LPS stimulated co-cultures the role of this protein in the stimulation of chemokine induction was assessed using ICAM-1 blocking antibodies, which were added to the MLE-12 cells prior to incubation with MPI cells. Under these conditions, only MCP-1 production was reduced, suggesting a specific role of ICAM-1 in the induction of this chemokine, but not the others tested (**Figure 8B**).

### MLE-12 cells are the main cellular sources of MCP-1 in LPS stimulated co-cultures

**Figure 9:**
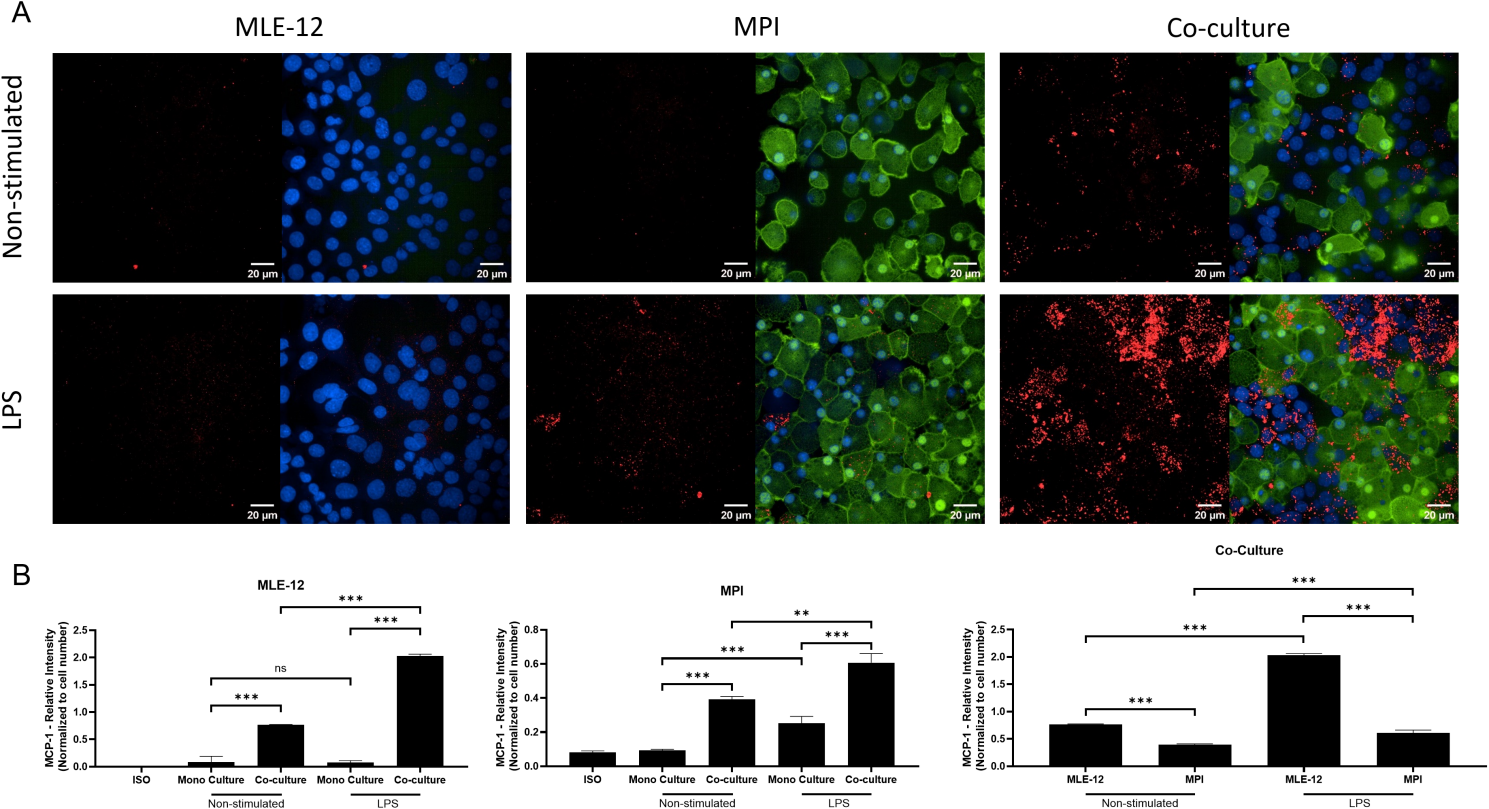
MLE-12 cells are the dominant source of MCP-1 in co-culture model. Intracellular cytokine staining of MCP-1 in MPI and MLE-12 cells grown isolated or in co-cultures, when left unstimulated or exposed to LPS **(A)**. Quantification of mean relative fluorescence intensity (RFU) per cell of intracellular MCP-1 **(B)**. hCD68^GFP^ MPI cells were used to discriminate MPI from MLE-12 background using GFP expression (green) and cell nuclei were counterstained with Hoechst 33342 (blue). Images and data are representative of three experimental replicates, with MCP-1 relative spot intensity for the well expressed as mean ± SD for three biological replicates. Statistical significance was determined by Prism software using the One-Way ANOVA followed by Dunnett’s *post hoc* test. **p-value < 0.01; ***p-value < 0.001. ns was used to indicate when the differences were found to be non-significant.

To test which cell type was responsible for the overproduction of MCP-1 in the co-culture system, we used immunostaining and confocal microscopy and assessed the contribution of MLE-12 and MPI cells to MCP-1 production. We found that in mono-cultures unstimulated cells did not produce MCP-1 and LPS stimulated MPI but not MLE-12 cells were significant producers of MCP-1 in (**Figure 9**). In co-culture conditions, unstimulated MLE-12 cells produced significant amounts of MCP-1 and this was higher than the levels of this cytokine in MPI cells (**Figure 9**). Thus, while both cell types are cellular sources for the increased MCP-1 production in LPS-stimulated co-cultures, MLE-12 cells are a more potent source of this cytokine, driving the level increase.

## Discussion

In this study, we investigated how the interaction between alveolar epithelial cells and lung alveolar macrophage-like cells influences innate immune responses to bacterial endotoxin. Using a co-culture system of MLE-12 alveolar epithelial cells and MPI alveolar macrophage-like cells—a self-renewing, non-transformed cell line that models key features of alveolar macrophages—we demonstrated that cellular cross-talk significantly enhances proinflammatory responses to LPS compared to mono-cultures. Earlier reports have shown that epithelial– alveolar macrophage-like cell interactions modulate innate immune activity in the lung, however, specific mechanisms remained largely unexplored (29–31).

One of the most striking observations in our studies was the pronounced increase of cytokine and chemokine production to TLR4 and TLR2 ligand stimulation in co-culture. These data are in line with increased cytokine production obtained in an AM-epithelial cell model stimulated with unorganic and organic particles (29). Several mechanisms can explain the LPS induced enhanced cytokine production in our co-culture model. We found that MLE-12 cells produce LBP, a molecule essential for TLR4-mediated AM and MPI alveolar macrophage-like cells activation (18). Thus, the epithelial cell-derived LBP provides a physiologically relevant factor, enabling effective LPS recognition and downstream signaling. This mechanism aligns with *in vivo* studies showing that LBP is endogenously produced in the lung and is critical for host defense against Gram-negative bacteria (32, 33).

However, the amplification of inflammatory responses in the co-culture system could not be solely attributed to LBP. Enhanced cytokine secretion was also observed when using TLR2 agonists such as FSL-1 and cockroach extract, which are LBP-independent stimuli (25, 34). Furthermore, enhancement of LPS induced cytokine and chemokine production was observed in the presence of exogenous LBP as well. These findings point to LBP-independent mechanisms in the co-cultures that act in addition to LBP production.

The activation of NF-κB and MAP kinases play crucial roles in LPS induced cytokine production (35, 36). We found that in LPS stimulated co-cultures the activation of MAP kinases and NF-κB are enhanced in MPI alveolar macrophage-like cells and MLE-12 cells, respectively.

To explore these mechanisms further, we tested whether soluble factors from epithelial cells alone were sufficient to induce enhanced cytokine production in MPI alveolar macrophage-like cells. Conditioned media from MLE-12 cells failed to replicate the co-culture effect, indicating that direct cell-to-cell interaction was necessary. Interestingly, when MLE-12 cells were first exposed to LPS-activated MPI-conditioned medium and then co-cultured with naïve MPI cells, they were able to induce chemokines such as MCP-1, MIP-2, and IP-10 even in the absence of further stimulation. This observation suggests a feed-forward loop in which initial macrophage activation can prime epithelial cells to become pro-inflammatory themselves, subsequently stimulating further chemokine responses through contact-dependent signaling.

An important mediator of lung epithelial cell – macrophage interactions appears to be TNF-α. Secreted by MPI cells in response to LPS, TNF-α was sufficient to induce both apoptosis and ICAM-1 expression on MLE-12 cells. TNF-α–induced apoptosis is well documented in other epithelial systems (37–39) including MLE-12 cells (39), and its ability to induce ICAM-1 on type I alveolar epithelial cells has been shown as well (40, 41). TNF-α can TNF-α activate NF-κB and MAP kinases (42) consistent with our findings. To our knowledge this is first incidence of TNF-α–induced ICAM1 in the type II MLE-12 cells. ICAM-1 is known to bind LFA-1 on leukocytes to mediate their migration through blood vessels (43, 44). Nevertheless, in our system, it appears to enhance MCP-1 production in epithelial cells. This conclusion is supported by the observation that ICAM-1 blockade specifically reduced MCP-1 production in the co-culture, while other cytokines such as MIP-2 remained unaffected. Together with the TNF-α mediated overexpression of ICAM-1 in MLE-12 cells that we also show here these findings suggest that ICAM-1 plays a selective role in epithelial–macrophage communication and MCP-1 production. ICAM-1 expression is induced by NF-κB activation in cells. Consistent with this, our findings indicate the requirement for TNF-α in ICAM1 upregulation and also the activation of NF-κB in MLE-12 cells in LPS stimulated co-cultures. MCP-1 plays key roles in attracting activated leukocytes during inflammation and alveolar epithelial cells are an important and active source of MCP-1 in the lung during inflammation (45). Our studies, to our knowledge the first time, suggest an important contribution for macrophage -epithelial cell interactions and ICAM-1 induction in this process. Our data are consistent with ICAM-1–dependent, contact-mediated signaling. While antibody mediated ICAM-1 crosslinking has been shown to trigger downstream signaling in airway epithelial cells (46), naturally occurring ICAM-1 mediated signaling in epithelial cells has not been shown yet.

Taken together, our findings highlight the importance of epithelial–macrophage interactions in shaping pulmonary immune responses. The TNF-α–ICAM-1–MCP-1 axis may represent a conserved pathway for inflammatory amplification in the lung; however, further *in vivo* and patient-based studies will be required to determine whether these mechanisms operate across disease contexts such as pneumonia, asthma, acute lung injury, and pollutant exposure where TLR2 and TLR4 ligands play important roles (47–49). Moreover, the observed epithelial cell apoptosis raises important questions about how excessive immune activation may compromise barrier function, potentially exacerbating tissue damage or susceptibility to secondary infection (50–52). The overall picture is one of a tightly coordinated immune interface, with feedback loops and physical interactions modulating the magnitude and character of the response.

This study has several limitations. We used a transformed lung alveolar epithelial cell line (MLE-12) and a primary, GM-CSF-dependent fetal liver–derived macrophage model of alveolar-like macrophages, both of which differ from their *in vivo* counterparts. In addition, the system relies on relatively strong stimulation with defined TLR ligands. The use of complementary *in vitro* and *in vivo* models will therefore be important to validate and extend the mechanisms described here.

By dissecting the individual contributions of soluble factors, direct contact, and surface molecules like ICAM-1, this study provides mechanistic insight into how innate immunity is orchestrated at the epithelial surface. The co-culture model described here offers a tractable and scalable platform to investigate complex immune interactions and may serve as a valuable tool for drug screening and disease modeling in pulmonary immunology in future studies.

## Data Availability

The raw data supporting the conclusions of this article will be made available by the authors, without undue reservation.
