# Supplementary material for: Lung epithelial and alveolar macrophage-like cell interactions significantly modify innate responses to bacterial endotoxin with the involvement of direct cellular contacts, TNF-α, ICAM1 and MCP-1
Source: Front Immunol. 2026 Jan 9;16:1715943. doi: 10.3389/fimmu.2025.1715943 (PMC12827152; doi:10.3389/fimmu.2025.1715943)
Supplement: Supplementary file 1 [file DataSheet1.pdf]

Lung epithelial and alveolar macrophage-like cell interactions significantly modify innate responses to bacterial endotoxin with the involvement of direct cellular contacts, TNF-α, ICAM1 and MCP-1

Connor Wood[1]#* £ , Shagun Khera[2]#, Minjeong Woo[1] £ , Vikram Sharma[2], Justyna Lopatecka[2]€, Frederic Coulon[3], Zaheer Nasir[3], Vincent Delorme[1]$, Simon K Jackson[2], and György Fejer[2]*

1. Institut Pasteur Korea, Tuberculosis Research Laboratory, Seongnam, Gyeonggi, 13488 Republic of Korea

2. School of Biomedical and Healthcare Sciences, Faculty of Health, Plymouth University, Plymouth, United Kingdom,

3. Cranfield University, Faculty of Engineering and Applied Sciences, Cranfield, MK43 0AL, UK

* Address correspondence to:

connor.wood@ip-korea.org or gyorgy.fejer@plymouth.ac.uk

# Contributed equally to this work

$ Present address:

Insol Co., Ltd. R&D Center, Hanam, Gyeonggi, 12918 Republic of Korea

£ Present address:

Institut Pasteur Korea, Antibacterial Resistance Laboratory, Seongnam, Gyeonggi, 13488 Republic of Korea

€ Present address:

Hirszfeld Institute of Immunology and Experimental Therapy, Polish Academy of Sciences, Wrocław, Poland

Supplementary materials

Supplementary Figures

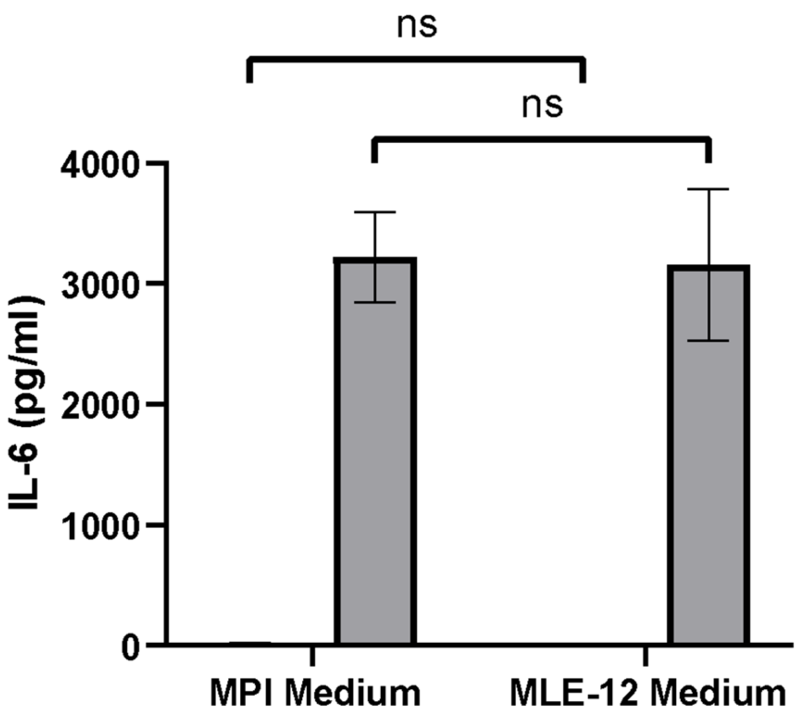

Supplemental Figure 1.

LPS stimulates cytokine production in MPI cells equally well when cultured in MLE-12 medium. Cells were seeded at a density of $5x10^5$/mL and stimulated with LPS and IL-6 secretions were quantified 24h post treatment via ELISA. Data are representative of three independent experiments (biological replicates) and expressed as mean ± SD. Statistical significance between MPI in MPI media and MLE-12 media was determined by Prism software using the unpaired Student's t-test. *ns* was indicated when the differences were found to be non-significant.

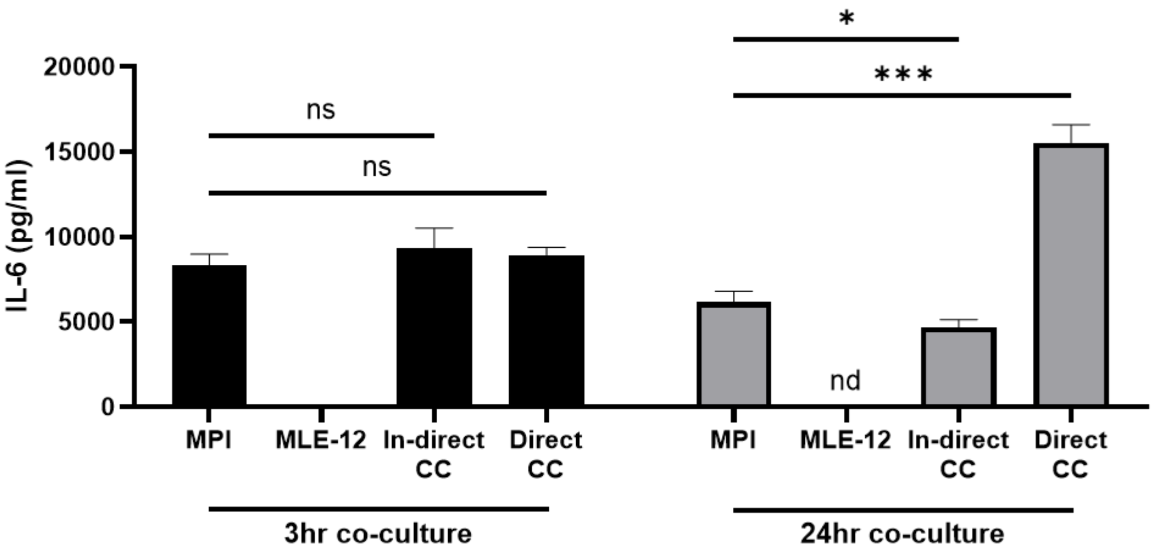

Supplemental Figure 2.

Time requirement for direct contact in the co-culture model to enhance LPS stimulated IL-6 production. MPI macrophages we added 3hrs and 24hrs prior to LPS stimulation and IL-6 secretions were quantified 24h post treatment via ELISA. Data are representative of three independent experiments (biological replicates) and expressed as mean ± SD. Statistical significance was determined by Prism software using the One-Way ANOVA followed by Dunnett's post hoc test. *, p-value < 0.05; ***, p-value < 0.001. ns was used to indicate when the differences were found to be non-significant.

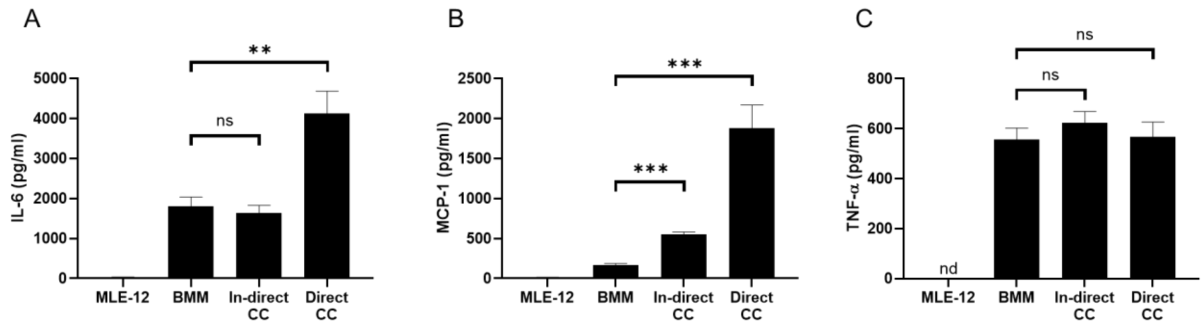

Supplemental Figure 3.

Co-culture of murine bone marrow macrophages (BMMs) and MLE-12 cells results in enhanced production of IL-6 and MCP-1 but not TNF-α upon stimulation. Production of IL-6 **(A)**, MCP-1 **(B)**, and TNF-α **(C)**, in response to 100 ng/mL LPS in MLE-12 and BMM cultures or co-cultures in the presence of serum. Cells were stimulated with LPS and cytokines were quantified 24h post treatment via ELISA. Data are representative of three independent experiments and expressed as mean ± SD for three biological replicates. Statistical significance between non-stimulated and LPS stimulated cells or between isolated cultures and co-cultures were determined by Prism software using the unpaired Student's t-test. **, p-value < 0.01; ***, p-value < 0.001. *nd*, not detectable. *ns* was used to indicate when the differences were found to be non-significant. nd was used to indicate no detectable value.

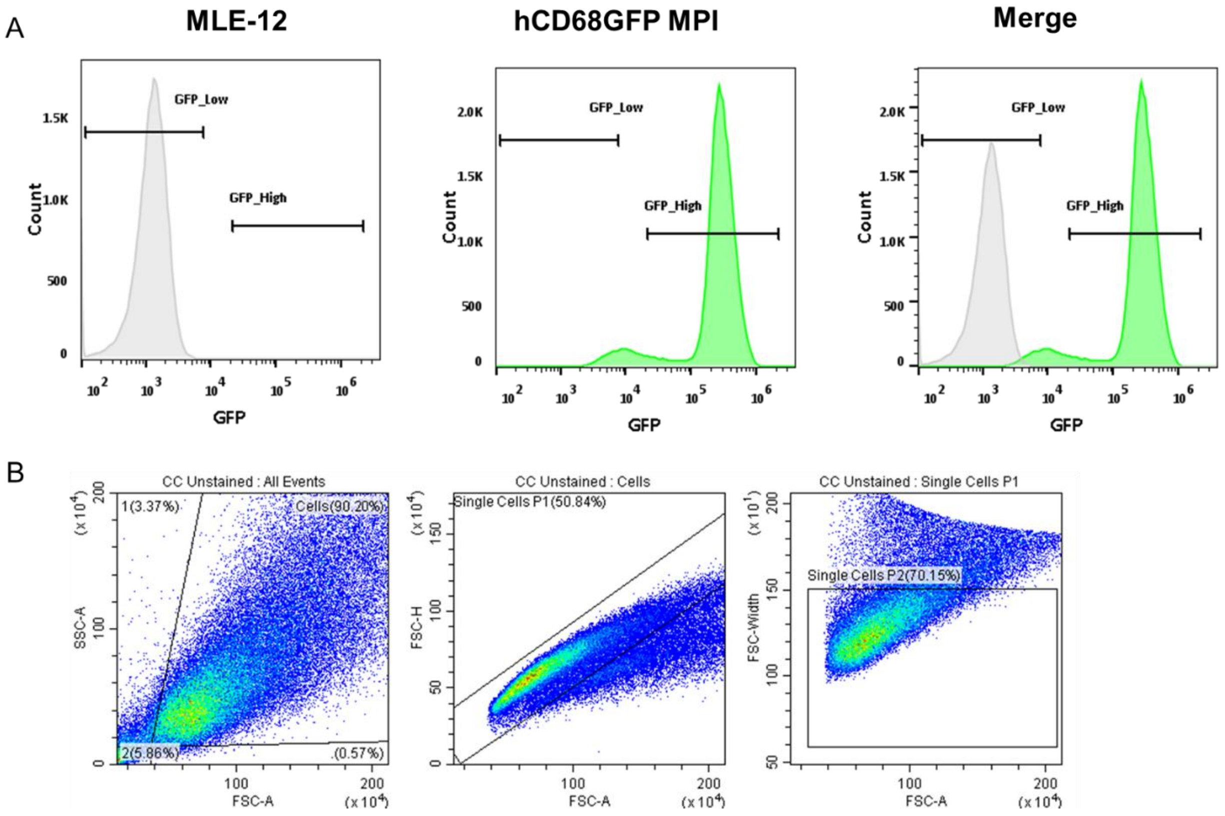

Supplemental Figure 4.

Example of flow cytometry cell gating techniques. Method for the discrimination of hCD^{68}GFP MPI and MLE-12 cell populations, when grown in co-culture. Cell populations were distinguished by high basal GFP expression level in MPI cells, gated has GFP^{high}, and absence of fluorescence in MLE-12 cells, gated as GFP^{low} (A). Method for isolation of single cell populations, cells were first identified via SSC and FSC, with following exclusion for doublets and clumps using FSC width and height (B).

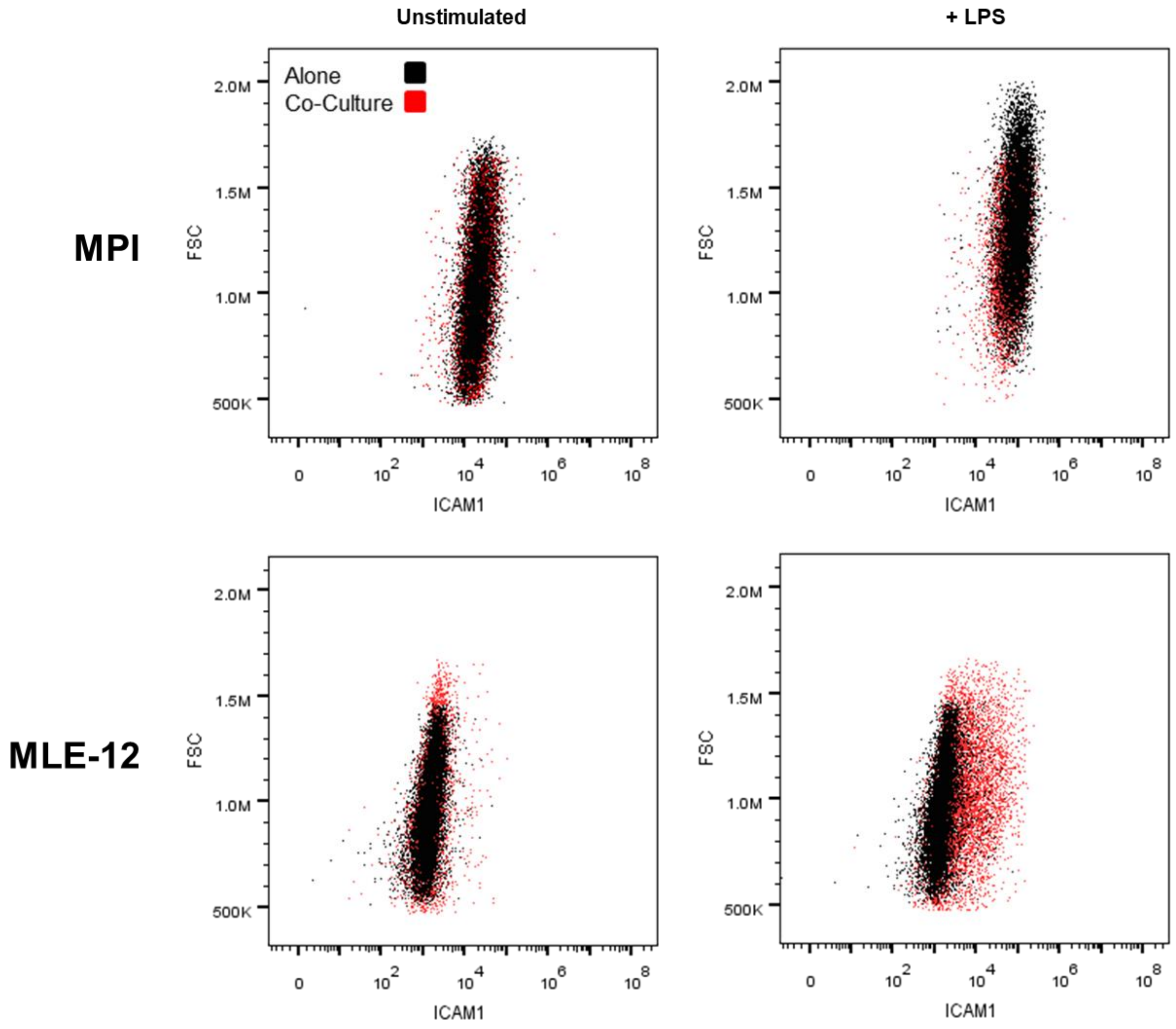

Supplemental Figure 5.

Corresponding scatterplots to ICAM1 expression on MPI and MLE-12 cells in mono- or co-culture during LPS stimulation. ICAM-1 expression levels in hCD68GFP MPI and MLE-12 cell cultures (Black) and co-cultures (Red) when stimulated with LPS. Cells were stimulated with LPS for 24h and stained for ICAM-1 expression, with cell populations being differentiated by constitutive GFP expression of MPI cells.

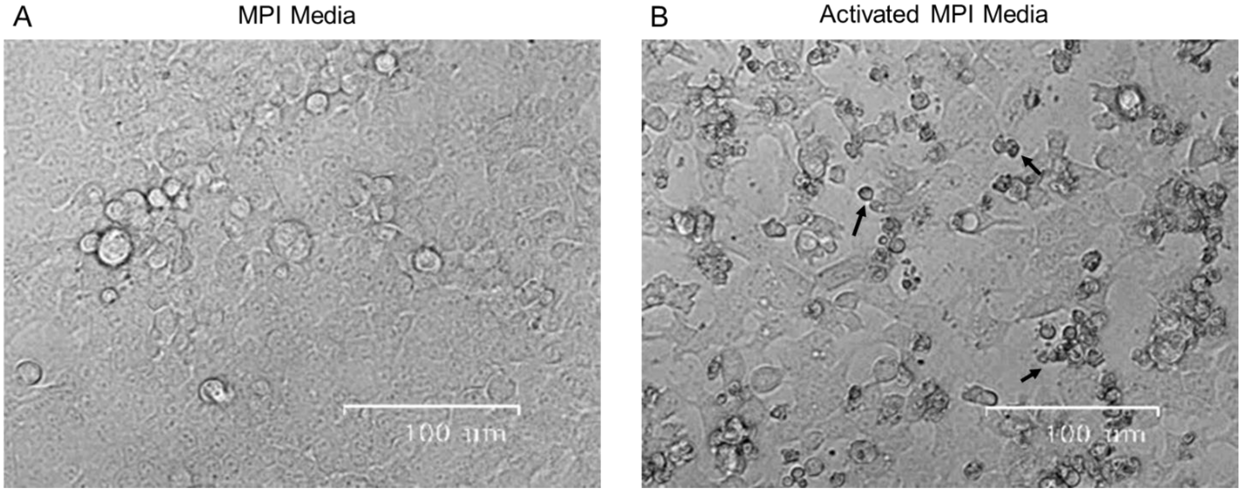

Supplemental Figure 6.

Visual observation of cytopathic effect (CPE) of LPS-activated MPI media on MLE-12 cells. MLE-12 cells were cultured with regular MPI cell conditioned media as a control **(A)** and LPS-activated MPI cell condition media **(B)** for 24h. CPE (Black Arrows) on MLE-12 monolayers was observed via light microscopy and captured using a ZOE Fluorescent Cell Imager system with a 20x objective.

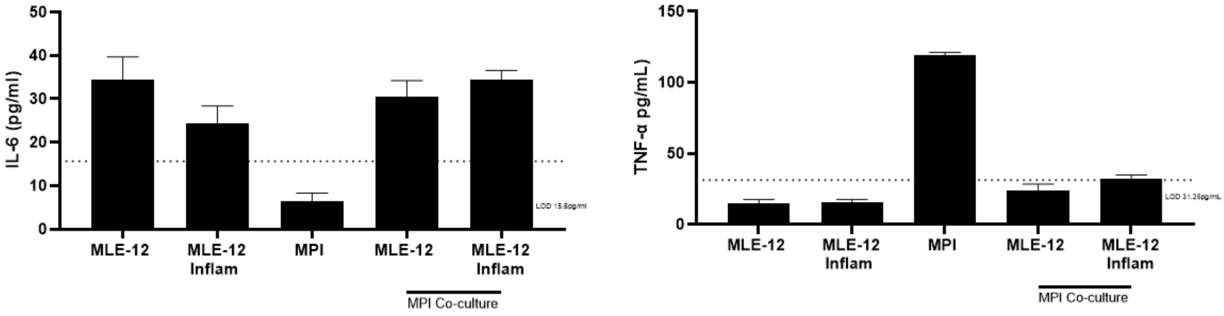

Supplemental Figure 7.

MLE-12 cells exposed to LPS-activated MPI cells supernatant do not in turn induce a cytokines response in naïve MPI cells, even in the absence of other inflammatory stimulants. Induction of IL-6 and TNF-α in co-cultures of naïve MPI cells with MLE-12 cells exposed to LPS-activated MPI cells supernatants, or inflammatory MLE-12 cells (MLE-12 Inflam), in the absence of additional external stimuli. Cells were cultured together and cytokines were quantified after 24h via ELISA. Data are representative of three independent experiments and expressed as mean ± SD for three biological replicates.
